# Can Parental Body Dissatisfaction Predict That of Children? A Study on Body Dissatisfaction, Body Mass Index, and Desire to Diet in Children Aged 9–11 and Their Families

**DOI:** 10.3389/fpsyg.2021.650744

**Published:** 2021-03-18

**Authors:** Natalia Solano-Pinto, Yolanda Sevilla-Vera, Raquel Fernández-Cézar, Dunia Garrido

**Affiliations:** ^1^Department of Psychology, University of Castilla-La Mancha, Toledo, Spain; ^2^Mathematics Department, Faculty of Education of Toledo, Castilla La Mancha University, Ciudad Real, Spain; ^3^Department of Personality, Evaluation and Psychological Treatment, Faculty of Psychology, University of Granada, Granada, Spain

**Keywords:** body dissatisfaction, childhood, family, drive for thinness, drive for muscularity

## Abstract

Body image has been associated with self-care and the assumption of either healthy habits or poor diets and eating disorders. As a vital element in the formation of a positive body image, the role of the family in childhood has been highlighted by a few studies. This study aimed to assess whether children’s body dissatisfaction could be predicted by their parents’ body dissatisfaction, body mass index (BMI), and approach to change. The sample consisted of 581 participants (366 parents and 215 children). The following instruments were used: anthropometric data, the Brief Scale of Body Dissatisfaction for Children, the IMAGE questionnaire (approach to change and drive for muscularity subscales), and the Eating Disorder Inventory-2 (body dissatisfaction and drive for thinness subscales). The results indicated that 19% of children, 22.8% of mothers, and 70.2% of fathers were overweight or obese. The multiple regression models developed for boys and girls explained 60 and 57% of the variance in body dissatisfaction, respectively. Several variables attributable to the mother (higher approach to change, higher drive for thinness, and higher BMI) and to the boys themselves (drive for muscularity, approach to change, and having a high BMI percentile) predicted a higher level of body dissatisfaction. For girls, only variables regarding themselves (approach to change, age, and BMI percentile) explained their body dissatisfaction. Relationships with the traits of the father were not detected for both models. The influence of sociocultural factors on the construction of gender and the negative consequences of mothers’ dieting for aesthetic purposes, on the development of children’s body image, are discussed.

## Introduction

Body image is developed mainly in childhood and adolescence and is formed by the experiential representation of one’s appearance and body shape (Smolak and Thompson, 2009). This representation may not match the objective and physical reality of the body because various biopsychosocial factors interact in the formation of the body image (Rodríguez and Alvis, 2015). From the cognitive-behavioral perspective, most previous studies agree that body image is the experience of one’s body and is a multifactorial construct where cognitive, emotional, perceptive, and behavioral aspects interact (Alleva et al., 2015; Longo, 2016). Cognitive-emotional issues are framed in an attitudinal dimension that includes thoughts and emotions related to focusing attention on the areas of the body that tend to be farther from the ideal (e.g., abdomen, hips), a drive for thinness and/or muscles, planned dieting and compulsive activity to change one’s figure, comparisons with slender bodies, fear of gaining weight, and emotional states, such as anger, sadness, and anxiety generated by dissatisfaction with one’s body (Trejger et al., 2015; Thompson and Schaefer, 2019).

Due to the mental representation of the body, individuals may feel satisfaction or dissatisfaction with their body. Dissatisfaction is associated with low self-esteem, anxiety, depression, and the risk of developing eating disorders and suicide in adolescence (Brausch and Gutierrez, 2009; Nayir et al., 2016). The obsessive internalization of a certain body and weight generates negative consequences for health. In this sense, a negative attitude toward the body promotes inappropriate behaviors—such as nutritionally incorrect behaviors, compulsive physical activity, and compensatory behaviors—to modify the body in both men and women (de Oliveira da Silva et al., 2018; Burnettea and Mazzeo, 2020) and is also a predictor of long-term weight gain (Lowe et al., 2019). As such, the prevention of body image disturbance has become a relevant issue in public health agendas (Kahan and Puhl, 2017).

The desire to have a certain figure is supported by the canons of beauty that, in Western society, have been associated with personal, social, and professional success. Among women, the socially transmitted canon has generated an image of an ideal body and has been pursued by many, including mothers who, in turn, are the reference points for their children. The sociocultural pressure on women has been so intense that many professionals postulate that body dissatisfaction is the “normative discontent” of the female sex, thus, unfortunately, normalizing the dissatisfaction women experience with their body; considering this normal for women, no measures are taken to decrease body dissatisfaction in this population (Carrard et al., 2018). Moreover, there is a tendency to incorporate the concept of being fit, and the importance of the musculature associated with a healthy body. In this sense, some authors find that incorporating muscular ideal in the ideal of a healthy body in women has negative consequences and leads to unhealthy behaviors (Uhlmann et al., 2018). These negative consequences extend past the woman’s own health. The woman as a mother is a key educational reference for her daughter in the development of a positive body image, and her attitudes toward her body affect the body dissatisfaction and eating behaviors of her daughter, as has been reported in the literature (Bauer et al., 2013; Arroyo et al., 2017; Zarychta et al., 2019). It has also been reported (Dahill et al., 2021) that adolescent children declared themselves to be affected by critical comments on their body and eating habits, or “fat-talk,” from their mothers.

Among males, there seems to be an increase in pressure toward a muscular and toned body that is associated with success (Karazsia et al., 2017; Arellano-Perez et al., 2019). Research shows that there is less body dissatisfaction among males, perhaps as a result of the influence of different reference models related to the male beauty canon, which reflects more heterogeneity than that of women. This is evident by how the results of different scales evaluating bodybuilding differed according to the social characteristics of the men evaluated (Cheng et al., 2016). In this respect, strategies to prevent body image disturbance are not oriented to young men, and there is scarce research on this subject (Doley et al., 2020). Nonetheless, fathers remain a significant role model for their children. Communication with both parents and affective closeness have implications on the development of body image and health in general (Hitti et al., 2020). However, the influence of the parents on children’s body image has been scarcely studied.

Pre-adolescence and adolescence are an evolutionary stage of vulnerability. Vulnerability regarding the body is not only due to neuropsychological and biopsychosocial changes, but also because body image acquires a significant role in the development of one’s identity (Rodríguez and Alvis, 2015; Vartanian and Hayward, 2020). In this sense, Wang et al. (2018) found that 72.8% of young female adolescents experienced body dissatisfaction and a drive for thinness versus 46.2% of males. Conversely, 14.6% of females and 40% of males strived for muscularity. Although this kind of study in childhood and pre-adolescence is sparse, the literature review reveals that approximately 50% of children aged 7–12 years want to be thinner (Vaquero et al., 2013). Similarly, Heidelberger and Smith (2018) found that among 10-year-olds, 60% of girls desired to be thinner versus 48% of boys; meanwhile, 62 and 50% of boys and girls, respectively, expressed a drive for muscles (Sánchez-Castillo et al., 2018). Although there are differences between boys and girls regarding body dissatisfaction, there has recently been a trend toward increasing dissatisfaction among young men (Holland and Tiggemann, 2016). Despite this, most research on body dissatisfaction focuses on young or adolescent girls and women (Dion et al., 2016; Paxton and Damiano, 2017).

One of the main issues studied in the field of body dissatisfaction has been the possible relationship between weight and body dissatisfaction. Additionally, obesity has traditionally been identified as a risk factor in the development of body dissatisfaction, primarily due to the social rejection of obesity and being overweight (Puhl and Heuer, 2010); this contributes to an obsessive desire for a particular physical figure (O’Brien et al., 2016). In this regard, a relationship was found between body satisfaction and body mass index (BMI) in a sample of more than 1000 adolescents (Kantanista et al., 2017), wherein male youths with low and normal weight experienced more satisfaction than those who were overweight; however, female youths with low weight experienced more satisfaction than those who were normal weight or overweight. Nonetheless, specific relationships between weight and body dissatisfaction have not always been found. For instance, Krch (2004) points out that being overweight and obese was not related to body dissatisfaction, thus emphasizing the greater importance of body perception relative to the BMI (Yan et al., 2015) and value attributed to the body (Shuanglong and Guangye, 2018). Regardless, the desire to modify one’s body is usually addressed by dieting to control weight; this gives rise to the prevalence of restrictive and compulsive behaviors in Western society (Puhl et al., 2015). Therefore, authors claim that a cognitive intervention based on facilitating the acceptance of one’s own body not only favors the development of a positive body image but also promotes the implementation of a balanced diet (Wilson et al., 2020).

One widely studied aspect is the role of the family in the formation of body image. The role of the family, along with the influence of peers and sociocultural factors, is incorporated in the tripartite model of body dissatisfaction as one of the main predictors (Thompson et al., 1999; Keery et al., 2004). They influence not only the development of body dissatisfaction but also that of eating disorders. The family influences the development of body image, like other forms of social learning, directly and indirectly (Bauer et al., 2017). Examples of direct influence are parents’ comments about the body shape and/or the need for weight control by children (Francis and Birch, 2005), while an example of indirect influence is the parents’ behaviors toward their own body (Cooley et al., 2008; Rodgers et al., 2009). Both types of influence may convey several factors, including the importance of the functionality of the body; care through a healthy, active, and shared lifestyle in the family; messages of affection and respect (Carbonneau et al., 2020) or, in contrast, body dissatisfaction (Arroyo et al., 2018).

Although many studies on family context and body dissatisfaction have focused on the mother–daughter relationship, several recent studies suggest the importance of the influence of both parents, specifically through critical comments on children’s bodies (Chng and Fassnacht, 2016; Wansink et al., 2017). Similarly, Biolcati et al. (2020) emphasizes that the influence of parents’ critical comments on the development of body dissatisfaction affects daughters in different life stages, such that mothers and fathers influence adolescence and young adulthood, respectively. Through qualitative and quantitative methods, McLaughlin et al. (2015) also studied dyads of 145 mothers and 145 daughters and concluded that there was an agreement between young women aged 8–12 years and their mothers on the negative influence of comments and teasing toward the body on the development of body dissatisfaction.

Studies carried out with children have evidenced the possible influence of parental body dissatisfaction on that of their children. As such, many studies have focused on linking eating behaviors and parental physical activity to the risk of being overweight in children (Matthews-Ewald et al., 2015). The authors found that, in a sample of children aged 3–7 years, parental dissatisfaction as assessed by a silhouette scale correlated with child dissatisfaction (Kościcka et al., 2016). Similarly, Webb and Haycraft (2019) highlight the link between body dissatisfaction and disadaptative eating habits of parents and the body dissatisfaction of children aged 6–9 years.

Although several studies have examined body dissatisfaction among children, most of the literature is focused on adolescent or adult women (Dion et al., 2016; Paxton and Damiano, 2017). The sociocultural pressure to achieve a thin body is present in Western society and, according to several authors, in most parts of the world (Izydorczyk and Sitnik-Warchulska, 2018). However, the sociocultural presence or influence of a drive for muscularity remains unclear. Moreover, the literature reveals that body dissatisfaction, behaviors to control weight, and maternal obsession with thinness influences the development of body dissatisfaction among daughters (Kluck, 2010; Neumark-Sztainerç et al., 2010; Bauer et al., 2013; Arroyo et al., 2017; Zarychta et al., 2019). Despite the rise in body dissatisfaction among boys, to the best of our knowledge, there are no studies analyzing mother–son dyads. Although the father’s role is crucial in the healthy development of children (Hitti et al., 2020), studies about paternal body dissatisfaction and its relationship with those of the children are scarce (Matthews-Ewald et al., 2015; Chng and Fassnacht, 2016; Kościcka et al., 2016; Wansink et al., 2017; Webb and Haycraft, 2019; Biolcati et al., 2020).

Given the crucial role of dissatisfaction in the health of young people and the role of the family as a major factor in the development of such dissatisfaction, this study examined whether a child’s body dissatisfaction was associated with and could be predicted by parents’ concerns about their weight and shape. Therefore, child–father and child–mother dyads were evaluated in the following variables: percentile of BMI, BMI, body dissatisfaction, drive for thinness, drive for muscularity, and beliefs about approach of modifying the body through diet. Specifically, the following hypotheses are proposed:

Hypothesis 1: The percentile of BMI, drive for muscularity, and approach to change are predictors of body dissatisfaction in boys and girls.

Hypothesis 2: Body dissatisfaction, drive for thinness, drive for muscularity, approach to change, and the BMI of the mothers and fathers are predictors of body dissatisfaction in boys.

Hypothesis 3: Body dissatisfaction, drive for thinness, drive for muscularity, approach to change, and the BMI of the mother and father are predictors of body dissatisfaction in girls.

## Materials and Methods

### Participants

This study was a cross-sectional study of children aged 8–11 years and their parents who were invited to participate in body satisfaction and healthy habits studies at the University of Castilla-La Mancha (Spain). Two hundred ninety children (8–11 years old) and their parents were invited to participate. From these, 215 (73.79%) child–father and/or child–mother dyads returned completed questionnaires. Thus, the final sample size was 581 participants (215 families, including 366 parents and 215 children). The inclusion criteria were: being enrolled in a primary school in Toledo (Spain) and willingness to participate in our study voluntarily and anonymously. All families (including both children and parents) completed a survey that assessed socioeconomic data; the measures are described below.

### Procedure

The school was informed about the research objectives and the requirement of voluntary and anonymous participation. Once the school agreed to participate, the families were asked to provide informed consent, and the anonymity of the data and voluntary participation was guaranteed. Through the school’s usual channels of communication, informed consent forms were sent to the families to be signed. Finally, the objectives of the research were explained to the children whose parents had given consent, and they were also informed about voluntary participation, and that participation could be terminated at any point.

Children were assigned a code that was also used for their families. The questionnaires for fathers and mothers were delivered through the children using an envelope. The parents returned the questionnaires in the sealed envelope and left them at the school office. The evaluation of the children was carried out collectively using the evaluation booklet where the socio-demographic data and the EDI-2 and IMAGE questionnaire appeared. Meanwhile, anthropometric measurements were taken individually, without informing the children. All data were collected anonymously, in such a way that the participants could not be identified. In turn, the database was safeguarded by researchers. The study was developed in compliance with the Helsinki Declaration regarding privacy, confidentiality, and informed consent, as well as the Data Protection Act enforced in Spain. The study also complied with the ethical requirements of the University of Castilla-La Mancha regarding research with humans.

### Measures

#### The Child’s Body Dissatisfaction

This was measured with the Brief Scale of Body Dissatisfaction for Children (EBICI; Baile et al., 2012). The psychometric properties of the instrument were provided by the referenced authors, who reported a reasonable internal consistency for Spanish participants (Cronbach’s *α* = 0.738). This scale consists of three items that measure body image. Each item has several options for the answer [e.g., Item 1: Regarding your physical appearance: (a) I think I have an adequate weight and image; (b) I would like to lose some kilograms; (c) I would like to lose many kilograms]. The participant is required to choose the one that best represents them; items 1, 2, and 3 range from 0–2, –1 – –2, and 0–3, respectively. The final score ranges from –1 to 7 and is the sum of the responses to all the questions, with a higher score associated with worse body satisfaction. The scale showed adequate internal consistency in our study (Cronbach’s α = 0.67).

#### Parent’s Body Dissatisfaction and Drive for Thinness

This was measured using the Spanish version (Garner, 1998) of the Eating Disorder Inventory (EDI-2; Garner, 1991). This questionnaire consists of 91 items that measure drive for thinness, bulimia, body dissatisfaction, ineffectiveness, perfectionism, interpersonal distrust, interoceptive awareness, maturity fears, asceticism, impulse regulation, and social insecurity. For this study, we used the subscales driven for thinness (e.g., I am worried because I would like to be a thinner person) and body dissatisfaction (e.g., I think my thighs are too thick). Both 6–points scales were rated from 0, meaning “never,” to 5, meaning “always.” For this study, the subscales driven for thinness and body dissatisfaction showed good internal consistency (Cronbach’s *α* = 0.87 for mothers and 0.71 for fathers, respectively). The final scores for the drive for thinness (nine items) and body dissatisfaction (seven items) subscales—ranging from 0–45 and 0–35, respectively—were the sum of responses to all the questions, where higher scores were associated with worse body satisfaction.

#### Parent’s and Children’s Parents and Children’s Drive for Muscularity and Approach to Change

This was measured with the Body Dissatisfaction Image questionnaire (IMAGEN; Solano-Pinto and Cano-Vindel, 2010; Solano-Pinto et al., 2017), which consists of 38 and 25 items in the original and abbreviated IMAGEN questionnaires, respectively (Solano-Pinto et al., 2017). For this study, we used approach to change of the cognitive-emotional subscale from the abbreviated version (three items, e.g., I should work on my diet). To measure drive for muscularity, three items evaluating concerns for the body were used (e.g., “I would like to have more muscle;” “I feel guilty when I cannot work out;” and “If I had more muscle, I would be more self-confident”). Each item was rated using a scale ranging from 0–4 scale, wherein 0 and 4 meant “never or almost never” and “always or almost always,” respectively. These subscales showed acceptable internal consistency in our study (Cronbach’s *α* = 0.74, 0.71, and 0.78 for children, mothers, and fathers, respectively).

### Data Analyses

First, because we were interested in whether body dissatisfaction differs between boys and girls, we tested both models separately. Second, we conducted descriptive statistics. Third, to investigate the relationship between a child’s body dissatisfaction and parents’ concerns about their own weight and shape (i.e., body dissatisfaction, drive for thinness, drive for muscularity, and approach to change), we computed bivariate Pearson correlations. Finally, to determine the unique influence of each predictor, we conducted multiple regression analyses using SPSS (Windows version 25), including the child’s demographics (age and BMI percentile) and parental BMI as control variables. For significant predictors, *f*^2^ was included as a measure of effect size. We considered an *f*^2^ of 0.02, 0.15, and 0.35 as small, medium, and large effects, respectively (Cohen et al., 2003).

To avoid multicollinearity and given that some of the measures could be interrelated, only significant correlates of a child’s body dissatisfaction were included as predictors in the subsequent multiple regression analysis. Moreover, to ensure that there was no multicollinearity among these predictor variables, we used the variance inflation factor (VIF). VIF values between and 1–10 are typically used to indicate the absence of multicollinearity (Cohen et al., 2003). Additionally, due to some missing data, we were concerned about potential variables that could be associated with these missing data and could bias our findings. To examine whether the missing at random assumption was satisfied (Little and Rubin, 2002), we conducted binary logistic regression to find additional potential predictors related to missingness.

## Results

Descriptive statistics are presented in Tables 1, 2.

**TABLE 1:** Demographic and clinical characteristics of our families (categorical variables).

	**Number**	**Percentage**
**Mothers**		
**Education**		
Low (no or primary education)	3	2
Medium (secondary education)	15	7
High (tertiary education)	135	63
**Home Country**		
Spain	196	91
Morocco	3	1
Other	7	3
**Race**		
Caucasian	90	95
African American	3	1
Other	2	1
**Occupation**		
Employed	163	76
Housewife	38	18
Student	1	0.5
**Marital status**		
Single	7	3
Married	175	81
Separated	18	8
Widowed	1	0.5
**Socio-economic level**		
Upper	23	11
Middle	90	42
Lower	29	13
**Fathers**		
**Education**		
Low (no or primary education)	56	26
Medium (secondary education)	13	6
High (tertiary education)	114	53
**Home Country**		
Spain	180	84
Morocco	3	1
Other	3	1
**Race**		
Caucasian	85	40
African American	3	1
Other	1	1
**Occupation**		
Employed	172	80
Housework	8	4
Retirement	1	0.5
**Marital status**		
Single	4	2
Married	174	81
Separated	10	5
**Socio-economic level**		
Upper	44	20
Middle	90	42
Lower	14	7

**TABLE 2:** Descriptive statistics for our families (continuous variables).

	**Mean**	***SD***	**Min-max**	**Range**	**Missing (%)**
Sons (*N* = 106)					
BMI percentile	50.47	30.64	1–99	–	1 (1)
Body dissatisfaction	1.03	1.88	–1 to 6	–1 to 7	0 (0)
Drive for muscularly	4.38	3.24	0–12	0–12	6 (6)
Approach to change	4.00	4.10	0–12	0–12	8 (8)
Daughters (*N* = 109)					
BMI percentile	57.45	31.38	1–99	–	0 (0)
Body dissatisfaction	0.85	1.73	–1 to 7	–1 to 7	0 (0)
Drive for muscularly	2.24	2.71	0–12	0–12	6 (6)
Approach to change	3.09	3.32	0–12	0–12	5 (5)
Mothers (*N* = 191)					
BMI (kg/m^2^)	23.25	3.52	17.65–40.35	–	24 (11)
Body dissatisfaction	16.76	8.08	0–38	0–45	16 (7)
Drive for muscularly	2.69	2.41	0–12	0–12	32 (15)
Drive for thinness	10.65	7.75	0–35	0–35	36 (17)
Approach to change	5.56	3.75	0–12	0–12	8 (4)
Fathers (*N* = 182)					
BMI (kg/m^2^)	26.65	3.17	20.06–38.09	–	33 (15)
Body dissatisfaction	14.23	6.41	0–40	0–45	39 (18)
Drive for muscularly	2.71	2.84	0–12	0–12	35 (16)
Drive for thinness	7.83	5.42	0–26	0–35	34 (16)
Approach to change	4.92	3.79	0–12	0–12	16 (9)

### Children’s Characteristics

From the sample, 49.3% (*n* = 106) were male, with a mean age of 9.78 years (range: 8–11 years). The majority of children (70.7%) were between the 10th and 85th percentile for BMI, considering the normal range. Only 41 (19.1%) had a percentile above the 85th, indicating that they were overweight or obese.

For both gender (*M_*daughters*_* = 9.74, *M_*sons*_* = 9.81; *p* = 0.60) and BMI percentile (*M_*daughters*_* = 57.45, *M_*sons*_* = 50.47; *p* = 0.10), results were not significantly different between daughters and sons.

### Mothers’ Characteristics

The mothers were 43.21 years old on average (range: 24–53 years). Most mothers were of normal weight; 69.8% showed a BMI under 25, and 3.1% a BMI under 18.5. Only 41 (19.1%) participants were overweight, while 8 (3.7%) were obese. The vast majority were Caucasian, married, had a high education level, and were employed.

### Fathers’ Characteristics

The fathers showed a mean age of 45.45 years (range: 28–63 years). Most of them (*n* = 123, 57.2%) were overweight, and 28 (13.0%) were obese. The vast majority were Caucasian, married, had a high education level, and were employed.

Before conducting the analyses, we tested for multicollinearity. No multicollinearity was evident among the tested predictors; this was evidenced by the VIF for the predictors, which ranged between 1.04 and 2.05, with tolerance values ranging between 0.45 and 0.90 (Cohen et al., 2003). Additionally, potential predictors of the missing data from those variables that correlated with a child’s body dissatisfaction were examined using binary logistic regression. The following results did not show any potential predictors among those evaluated: mother’s drive for thinness (*p* = 0.25), father’s approach to change (*p* = 0.83), mothers’ BMI (*p* = 0.70), and father’s BMI (*p* = 0.05).

### Body Dissatisfaction in Sons

Bivariate Pearson correlations analyzing the body dissatisfaction scores of the boys in the sample are presented in Table 3. Body dissatisfaction in the son was related to a higher son’s approach to change, son’s drive for muscularity, higher body dissatisfaction of the mother, higher drive for thinness of the mother, and approach to change of the mother.

**TABLE 3:** Pearson correlations between son’s body dissatisfaction with the other variables of interest.

		**1**	**2**	**3**	**4**	**5**	**6**	**7**	**8**	**9**	**10**	**11**
Son’s variables	(1) Body dissatisfaction	−										
	(2) Drive for muscularly	0.37*	−									
	(3) Approach to change	0.59*	0.47*	−								
Mother’s variables	(4) Body dissatisfaction	0.34*	0.09	0.23*	−							
	(5) Drive for muscularly	–0.01	–0.01	0.06	0.42*	−						
	(6) Drive for thinness	0.42*	0.18	0.30*	0.67*	0.58*	−					
	(7) Approach to change	0.20*	0.04	0.04	0.46*	0.41*	0.66*	−				
Father’s variables	(8) Body dissatisfaction	0.05	–0.06	–0.06	0.11	0.01	–0.03	0.14	−			
	(9) Drive for muscularly	–0.02	–0.20	–0.04	0.23*	0.26*	0.26*	0.14	0.06	−		
	(10) Drive for thinness	0.13	–0.07	–0.01	0.10	0.16	0.10	0.10	0.31*	0.50*	−	
	(11) Approach to change	0.17	0.07	0.04	0.20	0.17	0.22*	0.36*	0.32*	0.30*	0.59*	−

*p < 0.05.

For our main analysis, we conducted a multiple linear regression analysis with the sons’ body dissatisfaction as the outcome variable. The rest of the variables (i.e., son’s approach to change, son’s drive for muscularity, mother’s body satisfaction, mother’s drive for thinness, and mother’s approach to change) were included as predictors. The child’s demographics (age and BMI percentile) and parental BMI were included as controls in this analysis.

**FIGURE 1:**
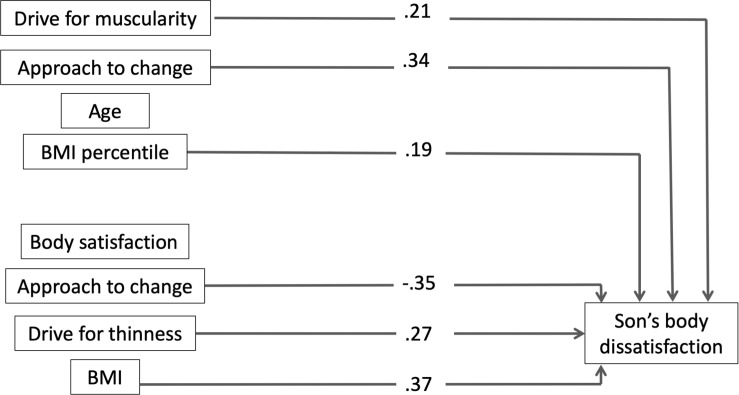
Results from multiple regression analysis for son’s body dissatisfaction. Only significant paths are displayed (*p* < 0.05) as β coefficients.

The results of the regression analysis are presented in Table 4 and Figure 1, including the standardized regression coefficients (βs) and the change in *R*^2^ for each predictor. The model accounted for 60% of the total variance in son’s body dissatisfaction (*F*[8,61] = 11.572, *p* < 0.001). Drive for muscularity, approach to change, mother’s approach to change, mother’s drive for thinness, BMI percentile of the son, and mother’s BMI, accounted for 14, 35, 4, 18, 24, and 11%, respectively, of the variability, wherein higher drive for muscularity, approach to change, mother’s drive for thinness, son’s BMI percentile, and mother’s BMI and lower mother’s approach to change, were related to worse body satisfaction of the son. The other predictors were not significant (*p* > 0.05). All predictors showed a medium effect size (mother’s drive for thinness: *f*^2^ = 0.22, son’s BMI percentile: *f*^2^ = 0.32, son’s drive for muscularity: *f*^2^ = 0.16, and mother’s BMI: *f*^2^ = 0.12), except for the mother’s approach to change (*f*^2^ = 0.04) and son’s approach to change (*f*^2^ = 0.54) which showed small and large effect sizes, respectively.

**TABLE 4:** Linear regression analyses to determine the influence of each predictor on son’s body dissatisfaction.

	**Son’s body dissatisfaction**					
	***B***	***SE***	***P***	***R*^2^**	**95% CIs**	
					**Lower bound**	**Upper bound**
Son’s approach to change	0.146	0.045	0.002	0.35	0.056	0.236
Son’s drive for muscularity	0.120	0.055	0.032	0.14	0.011	0.230
Mother’s body satisfaction	0.021	0.025	0.386	0.11	–0.028	0.070
Mother’s approach to change	–0.166	0.060	0.007	0.04	–0.286	–0.047
Mother’s drive for thinness	0.059	0.029	0.044	0.18	0.002	0.117
Son’s age	–0.258	0.160	0.112	0.00	–0.578	0.062
Son’s BMI percentile	0.012	0.006	0.029	0.24	0.001	0.024
Mother’s BMI	0.186	0.049	<0.001	0.11	0.088	0.283

### Daughter’s Body Dissatisfaction

Bivariate Pearson correlations of the daughter’s body dissatisfaction scores are presented in Table 5. Worse body satisfaction in the daughter was related to higher drive for muscularity, higher approach to change, worse mother’s body satisfaction, and higher mother’s drive for thinness.

**TABLE 5:** Pearson correlations between daughter’s body dissatisfaction with the other variables of interest.

		**1**	**2**	**3**	**4**	**5**	**6**	**7**	**8**	**9**	**10**	**11**
Daughter’s variables	(1) Body dissatisfaction	−										
	(2) Drive for muscularly	0.50*	−									
	(3) Approach to change	0.65*	0.43*	−								
Mother’s variables	(4) Body dissatisfaction	0.20*	0.00	0.11	−							
	(5) Drive for muscularly	0.06	–0.52	0.09	0.17	−						
	(6) Drive for thinness	0.31*	0.14	0.18	0.55*	0.40*	−					
	(7) Approach to change	0.07	–0.03	0.04	0.54*	0.27*	0.46*	−				
Father’s variables	(8) Body dissatisfaction	0.15	–0.12	–0.01	0.13	–0.03	0.13	0.17	−			
	(9) Drive for muscularly	0.19	0.21	0.13	0.14	0.30*	0.41*	0.30*	0.06	−		
	(10) Drive for thinness	0.17	0.05	0.02	0.20	0.18	0.43*	0.28*	0.31*	0.55*	−	
	(11) Approach to change	0.02	0.07	–0.10	0.16	0.15	0.25*	0.38*	0.24*	0.37*	0.60*	−

*p < 0.05.

For our main analysis, we conducted a multiple linear regression analysis with the daughter’s body dissatisfaction as the outcome variable. The rest of the variables (i.e., daughter’s drive for muscularity, daughter’s approach to change, mother’s body satisfaction, and mother’s drive for thinness) were included as predictors. The daughters’ demographics (age and BMI percentile) and parental BMI were included as controls in this analysis.

**FIGURE 2:**
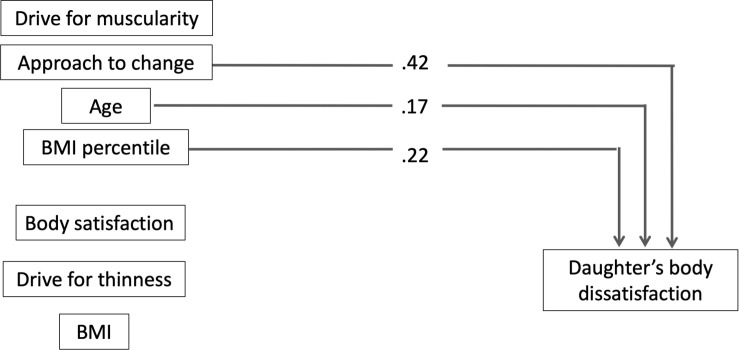
Results from multiple regression analysis for daughter’s body dissatisfaction. Only significant paths are displayed (*p* < 0.05) as β coefficients.

The results of the regression analysis are presented in Table 6 and Figure 2, including the standardized regression coefficients (βs) and the change in *R*^2^ for each predictor. The model accounted for 57% of the total variance in the daughter’s body dissatisfaction (*F*[7,67] = 12.555, *p* < 0.001). The approach to change, age, and BMI percentile of the daughter accounted for 43, 2, and 10%, respectively, of the variability, whereby a higher approach to change, higher BMI percentile, and being older were related to worse body satisfaction in the daughter. The other predictors were not significant (*p* > 0.05). All predictors showed a small effect size (daughter’s BMI: *f*^2^ = 0.10, daughter’s age: *f*^2^ = 0.00), except for the daughter’s approach to change, which showed a large effect size (*f*^2^ = 0.75).

**TABLE 6:** Linear regression analyses to determine the influence of each predictor on daughter’s body dissatisfaction.

	**Daughter’s body dissatisfaction**					
	***B***	***SE***	***P***	***R*^2^**	**95% CIs**	
					**Lower bound**	**Upper bound**
Daughter’s drive for muscularity	0.128	0.068	0.063	0.25	–0.007	0.264
Daughter’s approach to change	0.224	0.053	<0.001	0.43	0.1117	0.330
Mother’s body dissatisfaction	0.014	0.022	0.527	0.04	–0.030	0.058
Mother’s drive for thinness	0.048	0.025	0.063	0.09	–0.003	0.098
Daughter’s age	0.327	0.155	0.038	0.02	0.018	0.636
Daughter’s BMI percentile	0.012	0.005	0.014	0.10	0.002	0.021
Mother’s BMI	–0.042	0.041	0.311	0.01	–0.124	0.040

## Discussion

Different authors have emphasized the importance of body image in childhood and adolescent health (Kahan and Puhl, 2017); this was the motivation for this study. The general objective was to discover if parents’ body dissatisfaction can predict body dissatisfaction in their children in childhood: specifically, whether body dissatisfaction in girls and/or boys could be explained by their own approach to change and drive for muscularity and the body dissatisfaction, drive for thinness, drive for muscularity, and approach to change of their parents. For this, 215 (73.79%) child-father and/or child–mother dyads returned completed questionnaires, forming a final sample size of 581 participants with 215 families: 366 parents and 215 children. Anthropometric variables of the participants were analyzed using BMI.

For the body dissatisfaction of boys, 60% of the variance was determined by the model. The boys’ own significant variables were the BMI percentile, approach to change, and drive for muscularity. Among the parental variables, the maternal variables drive for thinness, body dissatisfaction, and approach to change significantly explained the body dissatisfaction of their sons, while no paternal variables were found to be statistically significant.

The model proposed to explain body dissatisfaction in girls accounted for 57% of the total variance in their body dissatisfaction. Among the variables of the girls, their BMI percentile, approach to change, and age were significant, while the drive for muscularity had a residual significance. Regarding the maternal variables evaluated, only drive for thinness had a residually significant explanatory character. None of the paternal variables explained their daughters’ body dissatisfaction.

Consequently, Hypothesis 1, which stated that the percentile for BMI, drive for muscularity, and approach to change are predictors of body dissatisfaction in boys and girls, is proven to a great extent. There were significant correlations between the dependent variable—body dissatisfaction—and BMI percentile, drive for muscularity, and approach to change for both girls and boys. In the model used to examine body dissatisfaction in boys, the BMI percentile, approach to change, and drive for muscularity were significant predictors; in the model for girls, drive for muscularity had only a residual significance and was not a remarkable predictor. These results confirm other studies’ findings; the relationship between weight and body dissatisfaction was emphasized along with being overweight as a risk factor for the development of body dissatisfaction (Puhl and Heuer, 2010; O’Brien et al., 2016), which takes place in both males and females (Kantanista et al., 2017) and may be confirmed in childhood. These data also reflect the existence of bodily dissatisfaction in children, confirming the increase in mean body dissatisfaction scores in men (Holland and Tiggemann, 2016; Karazsia et al., 2017).

The variable approach to change refers to the modification of weight through dieting. Therefore, we may state that thinking about dieting to change weight explains body dissatisfaction in both boys and girls. This may be in line with the increasing prevalence of restrictive and compulsive behaviors in Western society (Puhl et al., 2015). For future research, it would be appropriate to evaluate whether children with body dissatisfaction who are figuring out an approach to change also engage in restrictive and/or compulsive behaviors to modify the body, either with the desire to lose weight or become muscular. The Western canon of beauty is characterized by a slim body for women (Carrard et al., 2018) and muscularity for men (Karazsia et al., 2017). However, the canon is not static and has evolved to promote bodybuilding, as part of the concept of fitness, among women (Uhlmann et al., 2018). As such, in this study, both issues—the obsession with thinness and bodybuilding—were evaluated. One of the highlights of this study is that drive for muscularity explains body dissatisfaction significantly in boys and residually in girls. This is in line with studies highlighting that body dissatisfaction in children is linked to the desire for a different body, either slim or more muscular; this desire is present in adolescents—primarily in males, but also in females (Wang et al., 2018)—and in children (Heidelberger and Smith, 2018; Sánchez-Castillo et al., 2018). It should also be noted that the EBICI instrument used to assess body dissatisfaction evaluates, among others, the desire to lose weight, which would imply the desire for a thinner body. This data highlights that the children evaluated desire to have a muscular and slender body. Therefore, one may think that a slender body and a muscular body may be part of the ideal body internalized by children. This study cannot corroborate this hypothesis, but this may be considered in future research, along with an in-depth study of the canon of beauty and gender roles offered to boys and girls through different educational agents. These results should also be taken into account while formulating campaigns for obesity prevention and health promotion, to prevent body image disturbance and the approach of modifying the body through diet or physical activity, mainly in children who are overweight and obese. As some authors emphasize, the prevention of body image disturbance should be considered significant in public health (Kahan and Puhl, 2017), emphasizing a cognitive intervention that deepens the acceptance of one’s own body (Wilson et al., 2020).

Meanwhile, Hypothesis 2, which stated that body dissatisfaction, drive for thinness, drive for muscularity, approach to change, and the BMI of the parents are predictors of body dissatisfaction in boys, has been partially confirmed. Among the maternal variables evaluated, the variable with the greatest effect on the child’s body dissatisfaction was the mother’s BMI. These data would again confirm that being overweight and obese are predictive factors in the development of body dissatisfaction (Puhl and Heuer, 2010). However, both being overweight and having an overweight mother seem to be risk factors to be considered in the development of body dissatisfaction in children. This result should be interpreted in addition to the other significant variables. This may confirm the sociocultural pressure directed toward women, who aside from being female and overweight, and having an obsession with thinness, have no plan for weight change, which partly explains the child’s body dissatisfaction. Perhaps this may be interpreted by the desire for a socially accepted body contrary to that of their mother, who does not plan to change her body and is, in turn, socially rejected. It could be that the child internalizes these aspects and rejects the overweight status of their mother, who, although concerned about thinness, does nothing to alter her appearance; this rejection becomes tangible in the development of the daughter’s body dissatisfaction.

Some studies highlight an increase in mean body dissatisfaction scores in men (Holland and Tiggemann, 2016; Karazsia et al., 2017). This increase could be reflected by the prediction of children’s body dissatisfaction (Kościcka et al., 2016). However, in part, this was not verified in this study. In the evaluated sample, the mother’s dissatisfaction predicted the children’s dissatisfaction, as indicated in the literature (Bauer et al., 2013; Arroyo et al., 2017; Zarychta et al., 2019). Fundamentally, this prediction was made for sons, which is a novel finding of this study, and may be related to the above-mentioned increase in male scores in previous research.

Finally, Hypothesis 3, which stated that body dissatisfaction, drive for thinness, drive for muscularity, approach to change, and the BMI of the parents are predictors of body dissatisfaction in girls, was not confirmed, achieving a residual role in explaining body dissatisfaction in girls for the drive for thinness of the mother. This can be interpreted by the increased sociocultural pressure on the woman, causing the need to have a particular body associated with success to be internalized from an early age. This would explain why the variables evaluated in the girls, percentile of BMI, approach to change, and age, explained girls’ body dissatisfaction and not the variables evaluated in both parents.

The reviewed literature indicates that body dissatisfaction is common among women. It is worth focusing on its severity, despite its frequency, due to its negative health consequences (Carrard et al., 2018). Body dissatisfaction is related to the performance of nutritionally incorrect behaviors and compulsive physical activity, as well as unstable mental health (de Oliveira da Silva et al., 2018; Lowe et al., 2019; Burnettea and Mazzeo, 2020); additionally, as evidenced in this study, it has negative consequences for the sons. However, the variables evaluated in men have not been found to be predictive of body dissatisfaction in children. This has various interpretations. The traditional social role assigned to women makes them responsible for certain home tasks, such as those related to food, clothes, and health. Thus, mothers tend to spend more time with their children than fathers. Therefore, there is a higher prevalence of women as educational agents inside the family. In the evolutionary stage of childhood, the child may also have a greater bond with the mother, due in part to the greater time spent with her, while in other stages, such as in emerging adulthood, the father may have more influence (Biolcati et al., 2020). This result should be further explored in future studies since, as Doley et al. (2020) claimed, there are scarce studies that include males. In this sense, it would also be interesting to consider other variables in the family environment that may explain children’s body dissatisfaction, such as critical comments and teasing by parents (Dahill et al., 2021), communication style (Hitti et al., 2020), eating habits (de Oliveira da Silva et al., 2018; Webb and Haycraft, 2019; Burnettea and Mazzeo, 2020), and physical activity (Matthews-Ewald et al., 2015).

This study has several limitations that should be noted. The convenience sample and lack of control of the strange variables are highlighted. Future studies should include health indicators for both men and women that indicate whether the need for dieting could be due to health reasons. The performance of, type of, and reasons for diet may also be of interest. Additionally, a longitudinal design will allow the evolution of the predictors of body dissatisfaction in childhood and adolescence to be studied.

The suitability of the evaluation instruments used should be studied in depth since the EDI was created to evaluate aspects related to eating disorders, mainly in women. As such, it is necessary to research adequate instruments for the evaluation of body dissatisfaction in adults and children that take into account the multifactorial characteristics of the construct, rethinking the peculiarities of the canon of beauty that is currently transmitted to children. In contrast, an evaluation of the different types of values attributed to the body and their relationship to the construction of a positive body image may also be of interest. Finally, although the models presented do not fully explain children’s body dissatisfaction, they assert that other educational agents should be considered. Following the tripartite model of body dissatisfaction, the influence of peers and sociocultural factors should be examined (Thompson et al., 1999; Keery et al., 2004).

Despite these limitations, the study is novel since it includes the evaluation of the father and the assessment of the desire for bodybuilding in both sexes. Despite these limitations, the study is novel since it includes the evaluation of the father and the assessment of the drive for muscularity in both sexes. This enables the establishing of dyads of fathers, mothers, and children. Among the results, the predictive character of the mother’s body dissatisfaction on boys is notable.

The implications of these results should be considered when formulating education and health programs and administering them, not only for the prevention of dissatisfaction in childhood but also in adulthood. Sociocultural pressure on the body can contribute to the development of unhealthy behaviors, therefore attempts should be made to modify sociocultural models of the body. The normalization of corporal dissatisfaction as something expected for women must be removed from the collective imaginary, with actions carried out in different vital stages directed at the entire population. The influence of society as educational agents should be considered, where women and men should actively participate in the communication and education of their children (Hitti et al., 2020). This would increase health care and promote and encourage the positive development of their children’s body image. Additionally, women have different experiences—such as pregnancy, menopause, other unavoidable events, and the aging process—all of which produce changes in their physical appearance that must be socially valued and perceived positively to guarantee a positive construction of the body’s experience.

## Data Availability Statement

The datasets presented in this article are not readily available because individualized data from the project cannot be publically shared on a data repository due to the conditions of non-disclosure described in the consent form signed by the participants and their families. Requests to access the datasets should be directed to NS-P, natalia.solano@uclm.es.

## Ethics Statement

The studies involving human participants were reviewed and approved by the clinical drug research ethics committee “Complejo hospitalario de Toledo” (ref. 636). Written informed consent to participate in this study was provided by the participants’ legal guardian/next of kin.

## Author Contributions

NS, YS-V, and RF-C conceived the study and prepared the study materials. NS and YS-V collected the data. DG managed the data and conducted the analyses together with NS and RF-C. All the authors contributed to the interpretation of the results, provided critical revisions of the first draft, and approved the final version of the manuscript.

## Conflict of Interest

The authors declare that the research was conducted in the absence of any commercial or financial relationships that could be construed as a potential conflict of interest.
